# Left Pulmonary Artery from the Ascending Aorta: A Case Report and Review of Published Cases

**DOI:** 10.3390/jcdd8010001

**Published:** 2020-12-25

**Authors:** Rohit S. Loomba, Salvatore Aiello, Justin T. Tretter, Maira Gaffar, Jennifer Reppucci, Michael A. Brock, Diane Spicer, Robert H. Anderson

**Affiliations:** 1 Department of Pediatric Cardiology, Advocate Children’s Hospital, Oak Lawn, IL 60453, USA; 2 Department of Pediatrics, Chicago Medical School, Rosalind Franklin University of Medicine and Science, North Chicago, IL 60543, USA; salvatore.aiello@aah.org; 3 Cincinnati Children’s Hospital Medical Center, Cincinnati, OH 45299, USA; justin.tretter@cchmc.org; 4 Department of Pediatric Cardiology, University of Florida, Gainseville, FL 32611, USA; maira.gaffar@aah.org (M.G.); jennifer.reppucci@aah.org (J.R.); michal.brokc@aah.org (M.A.B.); spicerpath@hotmail.com (D.S.); 5 Department of pediatrics, Newcastle University, Newcastle Upon Tyne NE17RU, UK; sejjran@ucl.ac.uk

## Abstract

The left pulmonary artery arising from the ascending aorta is an infrequent finding. It may be found isolated or with intracardiac anomalies. We present a new case of the left pulmonary artery arising from the ascending aorta and pool these findings with those of previously reported cases. Associated cardiac, extracardiac, and syndromic findings are discussed along with the implications of these in the evaluation and management of this condition.

## 1. Introduction

The anomalous origin of one pulmonary artery from the aorta was first described by Fraentzel in 1868 [1]. Since then, it has been recognized that the anomalous origin can involve either the right or the left pulmonary artery. The second type, with anomalous origin of the left pulmonary artery, is much rarer, accounting for 0.03% of all congenital heart defects. It is often associated with other cardiac malformations [2,3]. The anomalous pulmonary artery, furthermore, can arise intrapericardially from the ascending aorta, or extrapericardially from the transverse or descending parts of the aorta. We present here a new case and review previously published cases describing anomalous intrapericardial origin of the left pulmonary artery from the ascending aorta.

## 2. Methods

PubMed, OVID, and Medline were queried using the following key words for the literature review: “anomalous origin of left pulmonary artery”, “left hemitruncus”, and “pulmonary artery anomalies”. The listed references of resulting articles were also used to identify additional reports. We then reviewed peer-reviewed publications, abstracts, and conference presentations for inclusion in our report. We excluded any cases that included origin of the left pulmonary artery arising from the transverse aorta, arterial duct, or descending aorta. We then analyzed the assembled data to reveal the associated malformations, the sidedness of the aortic arch, and clinical outcome.

## 3. Case Report 

### 3.1. Clinical History

Our patient, of female gender, was recognized during the fetal period when the mother was admitted to the antepartum unit at 34 weeks gestation because of pre-eclampsia. Monitoring the fetal heart demonstrated short bursts of non-sustained tachycardia up to 260 beats per minute. Fetal echocardiography demonstrated a large perimembranous outlet ventricular septal defect, with anterior malalignment of the supraventricular crest, albeit without significant obstruction of the right ventricular outflow tract. She had suffered two episodes of 1:1 atrioventricular conduction supraventricular tachycardia, each lasting less than 2 min, during the echocardiogram. No medical intervention for the tachycardia was deemed necessary at the time. She was delivered at 34 weeks gestation due to poorly controlled pre-eclampsia. Within two hours of delivery, she developed non-sustained supraventricular tachycardia associated with decreased perfusion and hypoxemia, with each episode, resolving spontaneously after 30 to 45 s. Vagal maneuvers were unsuccessful in terminating the episodes of tachycardia. After noting four such episodes, propranolol was initiated. 

**Figure 1:**
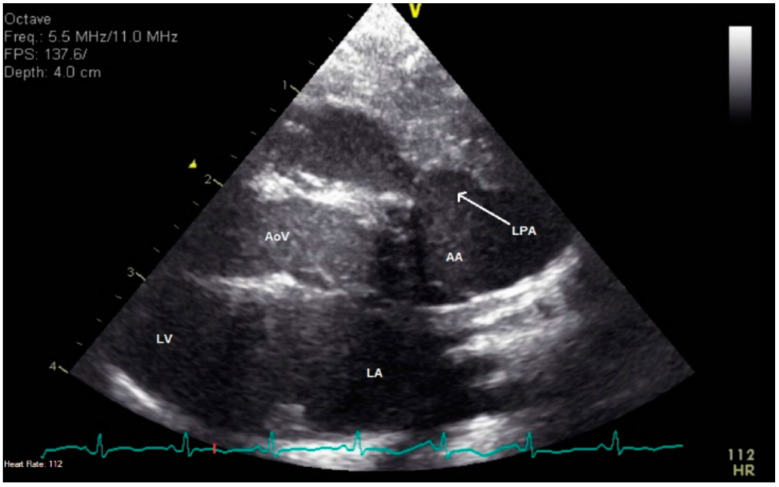
Two-dimensional echocardiography in the parasternal long-axis view. The aorta is seen arising from the left ventricle and there is a structure arising from the anterior aspect of the ascending aorta. This was confirmed by additional imaging to be the left pulmonary artery.

**Figure 2:**
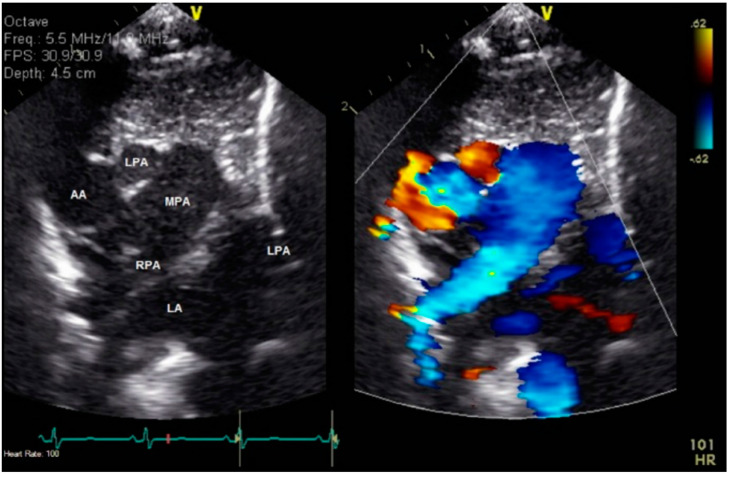
Echocardiography in the high-right parasternal short-axis view. The left panel is a two-dimensional image while the right panel is color interrogation of the same image. The pulmonary trunk is seen giving rise to a right pulmonary artery, without evidence of another vessel branching from the pulmonary trunk. The ascending aorta is visualized posterior and rightward, and there is an additional structure noted between the ascending aorta and the pulmonary trunk that appears to course anteriorly from the ascending aorta. This was confirmed by additional imaging to be the left pulmonary artery arising from the ascending aorta. The left pulmonary artery can be seen arising from the ascending aorta and then coursing leftward, crossing the pulmonary trunk and the right pulmonary artery in its course.

**Figure 3:**
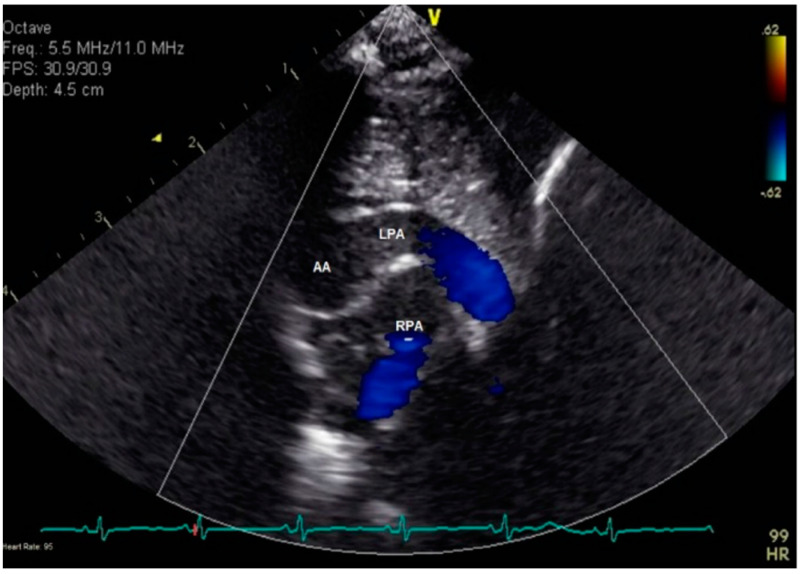
Echocardiography in the high-right parasternal short-axis view in a more superior plane than Figure 2. Color interrogation at this level demonstrates a vessel arising from the anterior aspect of the ascending aorta and then coursing leftward, passing over the pulmonary trunk and the right pulmonary artery.

**Figure 4:**
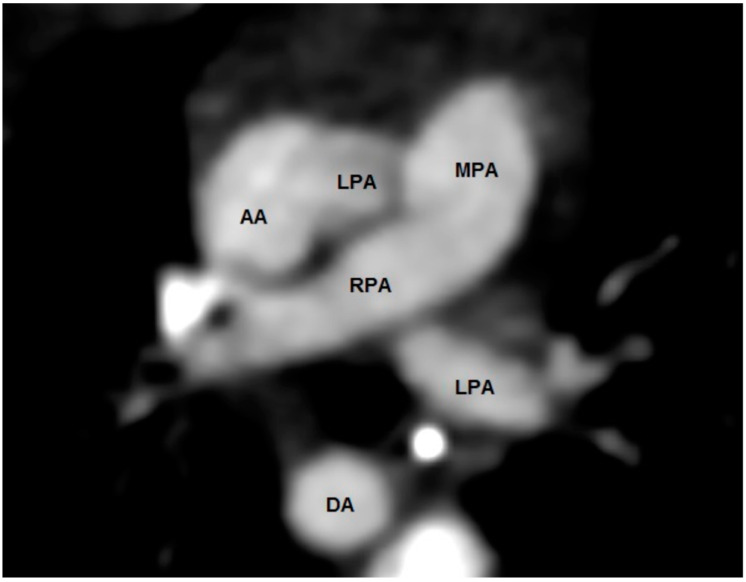
Computed tomography in the short-axis plane. The aorta is posterior and rightward to the pulmonary trunk. The pulmonary trunk gives rise to the right pulmonary artery, but not a left pulmonary artery. The left pulmonary artery arises from the anterior aspect of the ascending aorta, and then courses leftward, crossing the origin of the right pulmonary artery.

**Figure 5:**
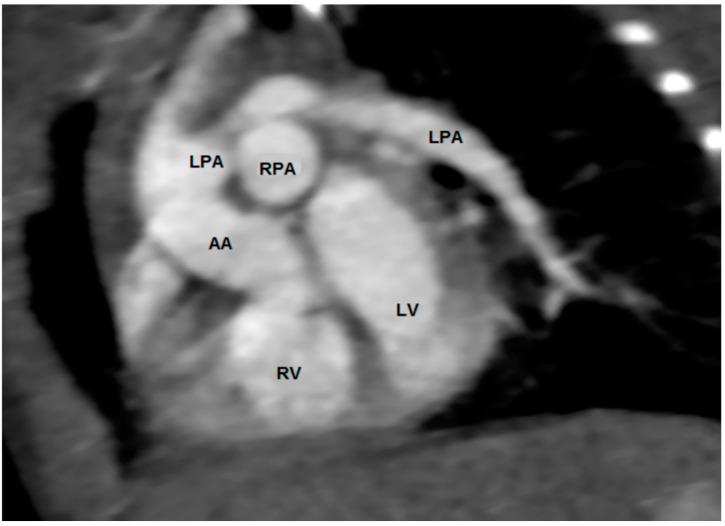
Computed tomography in the coronal plane. The left pulmonary artery arises from the ascending aorta and courses superiorly to cross the origin of the right pulmonary artery.

Echocardiography demonstrated tetralogy of Fallot with a large perimembranous outlet ventricular septal defect with anterior malalignment of the outlet septum, now with mild subpulmonary obstruction. The echocardiogram also revealed origin of the left pulmonary artery from the ascending aorta, with an aberrant left subclavian artery arising distally from a right-sided aortic arch (Figure 1, Figure 2 and Figure 3). Computed tomographic interrogation confirmed these findings (Figure 4 and Figure 5). The neonate did well from a cardiovascular standpoint, but unfortunately developed *E. coli* sepsis, with total necrotizing enterocolitis, and her parents chose to withdraw support.

Autopsy examination revealed the larynx, trachea, and bronchial tree to be normal. The lungs, however, were bilobed bilaterally, with an incomplete fissure between the right upper and middle lobes. The pulmonary parenchyma was congested, with consolidated areas scattered throughout all lobes.

**Figure 6:**
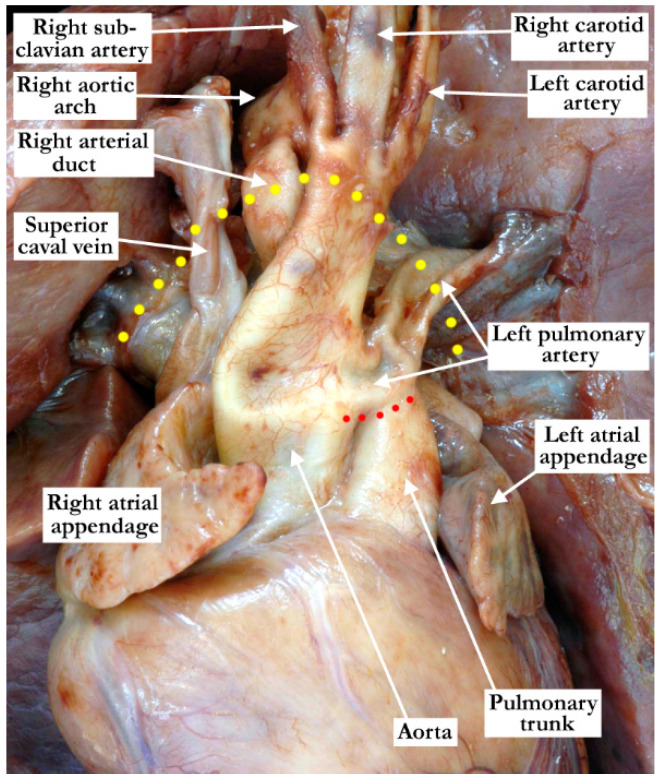
This anterior view of the aorta and pulmonary artery shows the origin of the left pulmonary artery from the ascending aorta as it crosses (red dots) the pulmonary trunk and the origin of the right pulmonary artery (not seen in this view). The aortic arch extends to the right and the arterial duct is also right-sided. The pericardial reflection is marked by the yellow dots.

**Figure 7:**
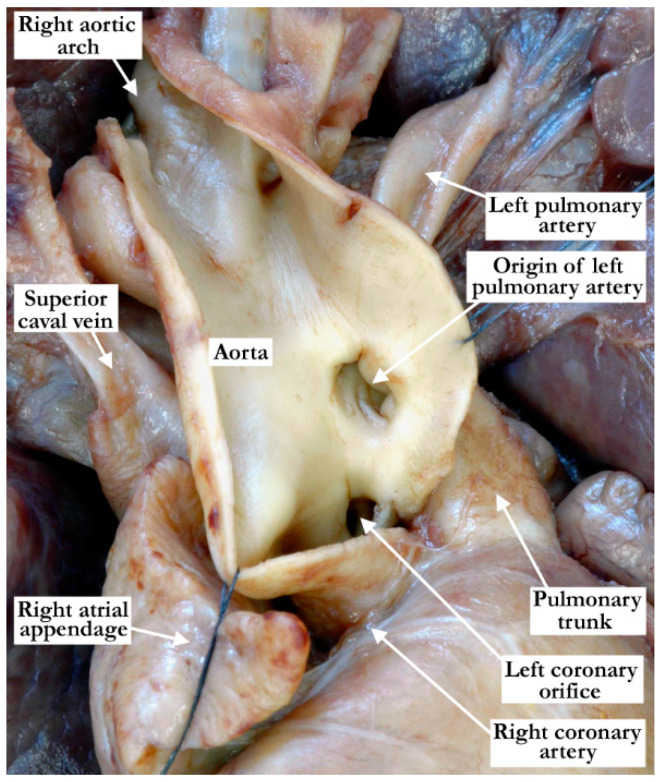
The right aortic arch and the ascending aorta have been opened to demonstrate the opening of the left pulmonary artery as it arises from the ascending aorta.

**Figure 8:**
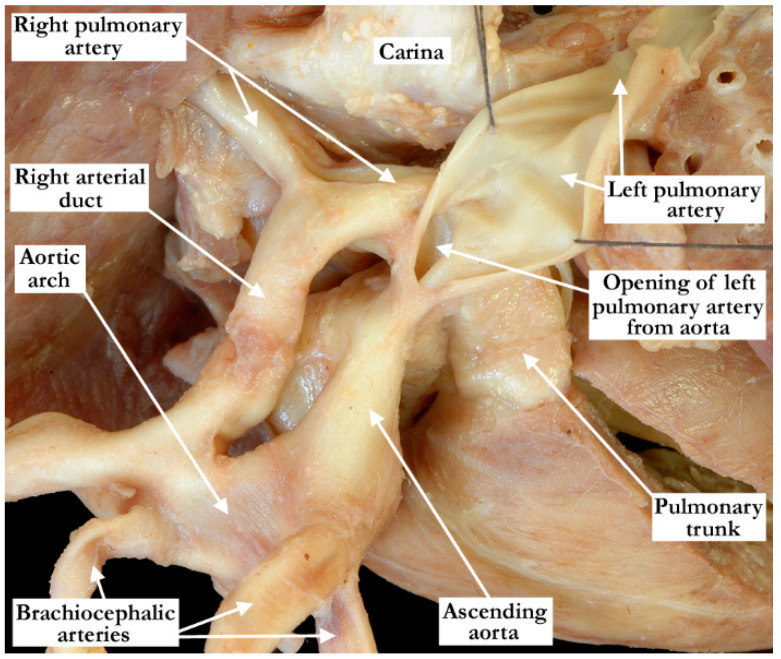
In this superior view, the aorta, right aortic arch and the pulmonary trunk have been folded forward and slightly rightward to show the origin of the left pulmonary artery from the ascending aorta and how it crosses the origin of the right pulmonary artery, which arose in the usual fashion from the pulmonary trunk. The patent arterial duct is easily appreciated extending between the right aortic arch and right pulmonary artery.

The heart showed usual atrial arrangement with concordant atrioventricular connections. The left brachiocephalic vein was absent, and there was a persistent left superior caval vein. The venoatrial connections, apart from the left superior caval vein, which drained through the enlarged coronary sinus, were otherwise normal. The oval foramen was probe patent, with a competent flap valve. The perimembranous outlet ventricular septal defect was of moderate size, with the aortic valve overriding the crest of the apical muscular septum. The outlet septum showed minimal anterosuperior deviation, but in the absence of obvious subpulmonary stenosis. The pulmonary trunk continued as the right pulmonary artery, with the left pulmonary artery originating intrapericardially directly from the ascending aorta. (Figure 6, Figure 7 and Figure 8). The left pulmonary artery, however, crossed the anterior aspect of the right pulmonary artery, giving the appearance known as crossed pulmonary arteries. The arterial duct was on the right, and extended from the right pulmonary artery to the right-sided aortic arch. The remaining anatomic features of the heart and arterial trunks were normal.

### 3.2. Review of Published Cases

Our analysis produced a total of 89 studies, which provided accounts of 125 cases of direct origin of the left pulmonary artery from the ascending aorta (Table 1). In Table 2, we list the described associated defects or malformations in these cases, having excluded 12 cases because of insufficient details [4,5,6,7,8,9]. Of the remaining 113 cases, 15 (13.3%) were not associated with intracardiac or extracardiac malformations [10,11,12,13,14,15,16,17,18,19,20,21,22,23]. Tetralogy of Fallot was the most frequently associated cardiac malformation, reported in 59 (52.2%) cases [2,3,24,25,26,27,28,29,30,31,32,33,34,35,36,37,38,39,40,41,42,43,44,45,46,47,48,49,50,51,52,53,54,55,56,57,58,59,60,61,62,63,64,65]. Of these cases, 9 also had so-called “absence” of the leaflets of the pulmonary valve [26,38,44,51,54,60,61]. When “pulmonary atresia with ventricular septal defect” is considered as a separate entity, a further 6 (5.3%) of the 113 cases exhibited this finding, although these were likely also examples of tetralogy of Fallot [17,66,67,68]. Double outlet right ventricle was noted in 3 (cases 2.7%) [69,70,71]. An isolated ventricular septal defect was reported in 12 (10.6%) cases [23,58,72,73,74,75,76,77,78]. Patency of the arterial duct was present in 16 (14.2%), with the duct being right-sided in 14 (87.5%) of these [3,17,24,30,33,60,69,79,80,81,82,83,84,85]. Of the 113 cases with sufficient anatomic details, an aberrant left subclavian artery was reported in 6 cases, and an aberrant right subclavian artery in 2 cases [3,24,33,68]. The only genetic condition reported in these cases was 22q11.1 deletion which was documented in 8 (7.1%) of cases [2,41,46,59,85,86]. The sidedness of the aortic arch was reported in only 74 cases, with 51 (68.9%) right arches and 23 (31.1%) left arches. Clinical outcomes were described for 98 patients. Of this group, there were 21 deaths reported (21.4%) (Table 3). We excluded 1 death from further analysis because of insufficient case details. Of the remaining 20 deaths, 11 (55.0%) followed surgical intervention. The average age of death was 20.2 months and the median age at time of death was 6 months. 

## 4. Discussion

When our patient is included, we are aware of 125 well-described examples of direct intrapericardial origin of the left pulmonary artery from the ascending aorta. Although obviously a rare disease, we believe the condition may be underreported. Its nomenclature has been variable and dynamic, contributing to the difficulty in identifying all reported cases. We encountered genuine examples described as “hemitruncus”, “pseudotruncus”, “truncus arteriosus communis type A3”, tetralogy of Fallot with unilateral absence of one pulmonary artery, and “aortopulmonary window type 2” [87]. Apart from the description of the lesion associated with tetralogy, the other terms should be avoided, since they do not accurately account for the observed anatomy. Others have commented on the difficulty in reviewing the literature due to the variability in origin of the pulmonary arteries, as well as the use of inconsistent nomenclature [88]. It is also likely that, in earlier eras, and even in the modern era, cases may have gone unrecognized because of diagnostic limitations [58,89,90]. It has been suggested that the overall group of patients with anomalous intrapericardial origin of a pulmonary artery can be divided into those without right ventricular outflow tract obstruction, who present earlier, and those with obstructed right ventricular outflow tracts, who present later [9]. Further separation can obviously be achieved by specifying anomalous origin of the right as opposed to the left pulmonary artery. While such an approach can be helpful, it is always best advised specifically to describe the findings in clear and concise terms. This helps avoid the ambiguity and confusion that can arise when advocating alphanumeric systems for classification.

Based on our recent experience from examination of normal development using high-resolution episcopic microscopy [91], we can now explain the abnormal finding. The developing outflow initially extends from the developing right ventricle to the margins of the pericardial cavity. At the pericardial margins, the single lumen of the outflow tract becomes confluent with the cavity of the aortic sac. The bilaterally symmetric arteries of the pharyngeal arches originate from the aortic sac, extending through the mesenchyme of the fourth and sixth branchial arches. Early in development, there is rotation at the margins of the pericardial cavity, permitting the developing aorta to connect to the rightward component of the aortic sac, while the developing pulmonary trunk connects to the leftward component. Concomitant with this rotation, a protrusion from the dorsal wall of the aortic sac grows into the intrapericardial outflow tract, serving as an aortopulmonary septum. This protrusion eventually fuses with the distal ends of the outflow cushions to divide the common outflow tract into the right-sided aortic channel and the left-sided pulmonary channel, at the same time closing a pre-existing aortopulmonary foramen [92]. Regression of the right sixth branchial arch is then necessary to separate completely the developing aortic and pulmonary channels. Failure of closure of the foramen results in aortopulmonary window [92]. Keeping these normal developmental processes in mind, we can now offer a developmental explanation for the situation in which the left pulmonary artery arises from the ascending aorta. The right and left pulmonary arteries themselves are formed within the pharyngeal mesenchyme, originating from the caudal walls of the arteries of the sixth pharyngeal arch [93]. Their origins are directly adjacent to the floor of the aortic sac. It is almost certainly unequal and abnormal partitioning of the aortic sac by the growth of the protrusion from its dorsal wall that provides an explanation for origin of either pulmonary artery from the intrapericardial part of the aorta [94]. Others have tried to explain the anomaly on the basis of persistence of an artery of the alleged fifth pharyngeal arch. Such an event, even if the fifth arch existed, would produce a channel that must be extrapericardial in nature. On this basis, the anomalous pulmonary artery would arise from the ascending aorta proximal to the origin of the brachiocephalic arteries, and would terminate in the dorsal aorta, or in the right or left pulmonary artery, having traversed the lumen of a persistently patent arterial duct. The fact that all described cases are intrapericardial rules out this option for development. 

**Figure 9:**
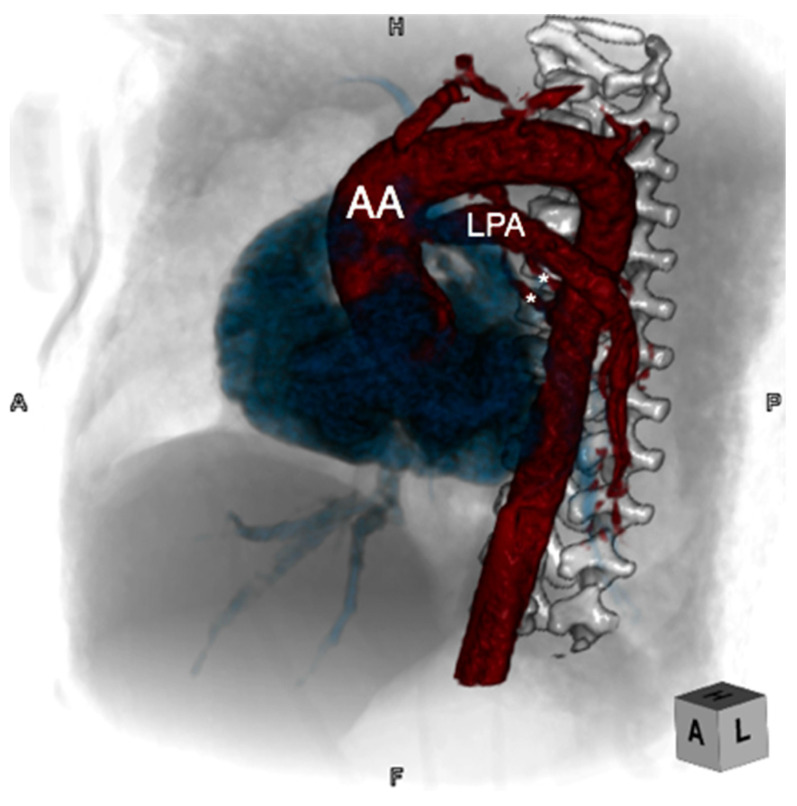
Computed tomographic three-dimensional (3D) reconstruction angulated in a left anterior oblique plane demonstrating an anomalous left pulmonary artery (LPA) arising from the ascending aorta (AA) in a patient with tetralogy of Fallot. Two small major aortopulmonary collateral arteries (asterisks) are demonstrating arising from the proximal thoracic descending aorta and coursing to the right lung.

**Figure 10:**
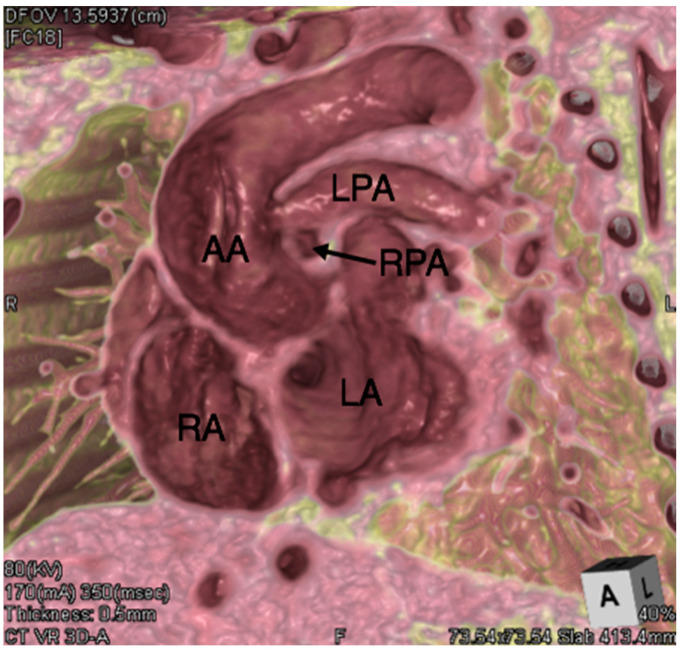
Computed tomographic 3D “virtual dissection” reconstruction in short axis of the atria demonstrating an anomalous left pulmonary artery (LPA) from the ascending aorta (AA) in a patient with tetralogy of Fallot. The right pulmonary artery (RPA) which arises from a hypoplastic pulmonary trunk is itself demonstrated to be hypoplastic. LA, left atrium; RA, right atrium.

In terms of clinical management, early recognition is vital. Our review identified an example of a misinterpreted image leading to failure of diagnosis of the anomalous origin of the left pulmonary artery in a patient with tetralogy of Fallot, with fatal consequences during reparative surgery [56]. Since that time, diagnostic capabilities have improved significantly. Computed tomography and echocardiography have been the initial screening tests for nearly all of our reviewed cases [72]. Magnetic resonance imaging has also been used to confirm the diagnosis, and to help in planning operative interventions [48,66,77,81]. Some cases may require multiple imaging modalities [71]. Even with advances in imaging, there are recent cases that have gone unrecognized subsequent to initial screening, only to be discovered during surgical intervention, or incidentally upon further imaging [58,76,89]. Multi-detector computed tomography is now the preferred method of multiple authors, who cite its increased reliability, efficiency, quality of images, and ability to provide an early diagnosis [58,60,67]. Advanced computed tomography techniques, such as newer three-dimensional modeling techniques and virtual dissection techniques, allow unique opportunities to further delineate the anatomy (Figure 9 and Figure 10).

**Figure 11:**
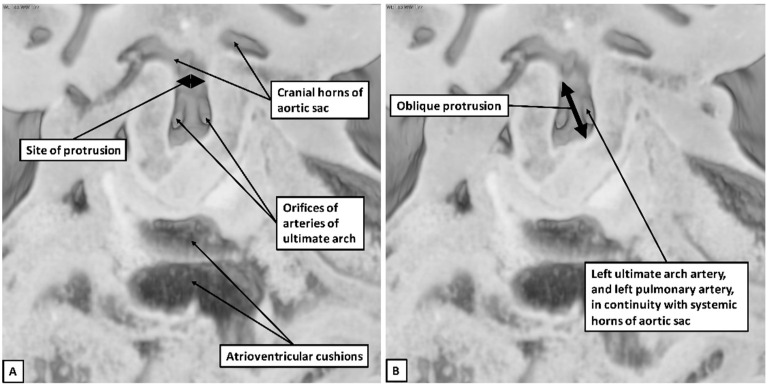
The panels show a possible explanation for anomalous intrapericardial aortic origin of the left pulmonary artery in the setting of a right aortic arch. Panel (**A**) shows the view of the aortic sac in a human embryo at Carnegie stage 13. At this stage, the cranial part of the aortic sac gives rise on each side to the arteries of the third and fourth pharyngeal arches, which will become the systemic arteries. The caudal part of the sac gives rise, again bilaterally, to the arteries of the ultimate pharyngeal arches, incorrectly described as the sixth arches since, as can be seen, there is no evidence of any fifth arch arteries. Normally the right-sided components of both the dorsal aorta and the ultimate arch artery regress, leaving a left-sided aortic arch and left-sided arterial duct. This requires formation of the aortopulmonary septum by growth of the dorsal wall between the cranial and caudal parts of the sac. The right hand panel (**B**) shows the consequence of growth of the dorsal wall, such that the origin of the left ultimate arch artery is incorporated into the systemic part of the sac, presuming that it is the left dorsal aorta and the left ultimate arch artery distal to the origin of the left pulmonary artery regress, leaving a right aortic arch and right-sided arterial duct.

The developmental considerations highlighted here are now demonstrated in Figure 11. In a review of 7329 patients diagnosed with congenital heart disease at a single institute, approximately 1% had some form of anomalous origin of the pulmonary artery from the ascending aorta. Only 0.03% of the total, however, consisted of anomalous origin of the left pulmonary artery [3]. This variant, overall, accounts for no more than one-third of cases of anomalous origin of a pulmonary artery from the ascending aorta, and is often reported to account for only one-tenth. Only one-eighth of all these cases exist in isolation, albeit isolated origin of the right pulmonary artery from the aorta being four-to-eight-fold more common [3]. Though a rarer malformation, anomalous origin of the left pulmonary artery has a higher association of accompanying cardiac defects [2]. We found tetralogy of Fallot, a right-sided patent arterial duct, and a right-sided aortic arch to be the most commonly occurring anomalies reported in the literature. Association with tetralogy of Fallot has been reported by others to be present in three-quarters of cases, higher than our estimate of half [3,42]. As with our review, others have noted the association with so-called “absence” of the pulmonary valve [38,44,51,54,61,86,95,96]. Overall, however, anomalous origin of the left pulmonary artery from the ascending aorta remains a rare malformation in patients having tetralogy of Fallot, reportedly seen in only 0.1% [55,97]. It is of note that tetralogy of Fallot and anomalous origin of the left pulmonary artery from the ascending aorta was believed to be a fatal combination beyond the first decade if left undiagnosed [42]. Multiple recent studies, nonetheless, have reported patients surviving beyond the first decade [3,19,33,52,57].

Other concomitant findings include a right-sided aortic arch, which has been reported in from half to three-quarters of all patients, in keeping with our review [3,40]. Association with a patent arterial duct is similarly consistent with our reported occurrence of 14.6% [3,12,40]. The variations of an anomalous left pulmonary artery arising from a patent arterial duct, however, should not be misinterpreted as true anomalous origin of the left pulmonary artery from the ascending aorta [37,96,98,99,100]. An aberrant subclavian artery has previously been reported in nearly half of one series, a frequency much greater than the result of our review [3]. This discrepancy may be explained by the small size of the previously reported series. A defect reported with less frequency is the presence of major aortopulmonary collateral arteries. Though rare, they provide unique challenges to the surgical correction and management of a patient [17,40,67,68]. The only associated genetic component noted was 22q11.1 microdeletion, reported in individual case studies [2,41,59,85,86]. The deletion remains a rare association, being documented in less than one-tenth of all reported cases.

Current studies report that if anomalous origin of either pulmonary arterial variant is left untreated, seven-tenths of patients will die from heart failure within 6 months, and four-fifths within 1 year [75]. In undiagnosed patients specifically, survival rate is no more than 30% beyond 1 year of life [75]. Several recent reports show that intervention can safely be performed on neonates and premature infants, eventually leading to complete resolution of symptoms [8,60,75,80,101]. The primary course of action after diagnosis, therefore, should be surgical correction unless precluded by comorbidities [59,63,80,101,102]. The most recent, and largest, case series demonstrates the high success rate of direct reimplantation [52]. In the absence of intracardiac defects, the procedure can be performed without the need for cardiopulmonary bypass, potentially resulting in fewer post-operative complications [15]. Restenosis across the anastomotic site of direct reimplantation is the leading cause for re-intervention [40,76,103]. In a recent series, the type of surgery did not significantly alter the long-term outcomes [60]. Reported survival at 20 years ranges from 80 to 92%, freedom from reoperation from 81 to 93%, and freedom from reintervention about 80% [60,75]. Though these reviews were not specific for anomalous origin of the left pulmonary artery, it is worth noting that no patients died in either report.

## 5. Conclusions

We conclude that anomalous origin of the left pulmonary artery from the ascending aorta is a rare defect. When present, it is often associated with other cardiac anomalies, most often tetralogy of Fallot. If left untreated, the prognosis is poor but surgical treatment is now routine with good outcomes.

## Figures and Tables

**Table 1:** Cases reported in the literature. AR(L)SA = Aberrant Right (Left) Subclavian Artery, CPB = Cardiopulmonary Bypass, DR = Direct reimplantation, DORV = Double Outlet Right Ventricle, MOF = Multi-organ failure, PAVSD = Pulmonary atresia ventricular septal defect, PLSVC = Persistent left superior vena cava, PVOD = Pulmonary Vascular Obstructive Disease, PVR = Pulmonary Valve replacement, MAPCA-RL= major aortopulmonary collateral vessels supplying the right lung, RL = Right lung, R-PDA= Right Patent Ductus Arteriosus, TOF = Tetralogy of Fallot, TR = Tetralogy of Fallot repair, VSD = Ventricular Septal Defect, * reported in Tagliente and Prifiti—no citation found [21,23]. Dy = days, mo = months, yr = years, wk = weeks, N/A = not available.

Year	Author	Age/Sex	Arch	Additional Defect	Surgical Procedure	Outcome	Follow Up
1941	Thomas	3 mo/F	--	TOF	None	Died	Died
1952	Sikl	Stillborn/M	Right	TOF	None	Died	Died
1964	Mudd	1 yr/M	Left	VSD	None	Alive	Alive at 4 yr
1964	Czarneck	4 yr/F	Left	TOF	TR	Died	Died intraop
1966	Weintraub	7 yr/M	Right	None-Isolated	DR no CPB	Survived	Alive
1969	Caudill	4.5 yr/F	Right	None-Isolated	DR no CPB	Survived	Alive at 3 yr
1970	Wyler	2 day/M	Right	TOF/APV	None	Survived	Alive at 2 mo
1971	Schiller	18 mo/F	Right	None-Isolated	DR	Survived	Alive at14 mo
1972	Morgan	20 mo/F	Left	TOF	DR, TR	Survived	Alive at 9 mo
1973	Herbert	15 mo/M	Right	R-PDA	DR no CPB, PDA ligation	Survived	--
1973	Verel	2 yr/F	Right	VSD, PS	--	--	--
	Verel	16 yr/F	Right	VSD	--	--	--
1973	Brill	3 mo/F	Right	R-PDA	None	Died	Infection
1974	Keane	4 mo/F	Right	TOF, bilobed RL	None	Died	Unknown
1975	Robin	5 mo/F	Left	TOF	Conservatively Managed	Died—9 mo	Pneum. meningitis
1975	Calazel	14 mo/--	--	TOF	TR	Died	--
	Calazel	--/--	--	TOF	None	N/A	--
1978	Sotomora	Newborn/F	Right	TOF, R-PDA, ALSA	None	Died	Postmortem
1978	Purcaro	43 yr/M	Left	TOF	--	--	--
1980	Calder	1 mo/M	Right	TOF/APV	None	Died	Pneumonia
	Calder	2 mo/F	Left	TOF/APV	None	Died	“Chest cold”
1981	Laborde	4 yr/	--	TOF	TR	Survived	--
1981	Duncan	22 mo/M	--	TOF	TR, DR	Survived	Alive
1982	Smallhorn	--	--	PAVSD, MAPCA-RL	--	--	--
	Smallhorn	--	--	PAVSD	--	--	--
	Smallhorn	--	--	None-Isolated	--	--	--
	Smallhorn	--	--	R-PDA	--	--	--
1984	Zinkovskii	--	--	TOF	TR, DR	Survived	--
1984	Nouri	1–5 yr/F		TOF	TR, DR	Survived	--
		1–5 yr/F		TOF	TR, DR	Survived	--
		6–12 mo/M		R-PDA	DR, PDA ligation	Survive	--
1985	Robida	4 yr/M	NR	TOF	TR	Died	PVOD
1987	Benatar	2.5 mo/F	Right	None-Isolated	DR	Survived	Alive
1987	Makhmudov	--	--	TOF	--	--	--
1988	Kutsche	8 Dy/F	Right	R-PDA, ASD, ALSA	--	--	--
	Kutsche	5 yr/M	Right	TOF, ALSA	--	--	--
	Kutsche	5 yr/F	Left	TOF, ARSA, PLSVC	--	--	--
1989	Fong	26 mo/NR	Right	TOF	DR, TR	Died	--
	Fong	3 mo/NR	Right	R-PDA, ALSA	DR, PDA ligation	Survived	--
1990	Gerlis	--	Left	--	--	--	Postmortem
1990	Sasaki	3 yr/F	--	TOF	DR, TOF repair	Survived	Alive
1990	Cherian*	5 yr/M	Right	TOF	TR	Survived	--
1991	Saxena	2 mo/M	Left	TOF	NR	Survived	--
1991	Endo	13 yr/F	Right	TOF	DR, TR	Died	PVOD
	Endo	26 yr/F	Right	TOF	DR, TR	Survived	Alive
1991	Sechtem	32 yr/F	Right	R-PDA	DR, PDA ligation	Survived	Alive at 6 mo
1993	Sreeram	1 Dy/--	Right	TOF/APV	Deferred Repair	N/A	Alive
1993	Mittal	20 yr/M	Right	None-Isolated	DR no CPB	Survived	Alive at 3 mo
1993	Py	12/F	Right	TOF	DR, TR	Survived	Alive 9 yr
1993	Prasad	23 yr/M	Left	None-Isolated	DR	Survived	Alive
1994	Bastos	--	--	--	Vascular Ring Repair	Survived	--
1995	Dodo	7 wk/M	Right	R-PDA, 22 q11.2 microdeletion	DR, PDA ligation	Survived	--
	Dodo	3 day/F	Right	R-PDA, 22 q11.2 microdeletion	DR, PDA ligation, interatrial closure	Survived	Constricted LPA-reoperation
1995	Lisbona	50 yr/F	--	--	--	--	--
1996	Tagliente	24 Dy/M	Right	None-Isolated	DR	Died	MOF
1998	Sulaimain	--	--	TOF, MAPCA-RL	Inoperable	--	--
	Sulaimain	--	--	--	--	--	--
	Sulaimain	--	--	--	--	--	--
	Sulaimain	--	--	--	--	--	--
1999	Wang	44 Dy/F	Right	DORV, VSD, R-PDA, facial dysmorphism	Banding of MPA, DR, PDA ligation	Survived	Alive, 33 mo DORV repair
1999	Saliba	1 mo/F		TOF, 22 q11 microdeletion	TR, DR	Survived	Alive at 6 mo
1999	Matsubayashi	3 mo/F	Left	VSD, RPA origin from descending Ao	VSD Repair, RVOT reconstruction, LPA and RPA anastomoses	Survived	Died post op Day 4
2000	Salaymeh	10 Dy/M	Right	R-PDA	DR without CPB	Survived	Alive at10 mo
2000	Soylu	14 yr/M	--	TOF	TR, DR	Survived	Alive at 6 mo
2001	Serr	34 wk-gestation/F	Right	VSD, ASD, APV, ALSA, 22 q11 microdeletion, interrupted aortic arch	VSD repair, ASD repair, DR, Interrupted aortic arch repair	Survived	Stenotic LPA at 6 mo
2001	Aru	3 wk/M	Right	None-Isolated	DR, without CPB	Survived	Alive at13 mo
2002	Amaral	40 Dy/F	--	TOF, absent thymus	DR, VSD closure with Dacron patch	Survived	Post-op Infection
2003	Prifti	37 Dy/--	--	VSD, CHF	DR, VSD repair	Survived	Alive at61 mo
	Prifti	34 Dy/--	--	None-Isolated	DR	Survived	Alive at27 mo
2004	Krishnamoorthy	10 yr/M	--	TOF/APV	--	--	--
2004	Santos	6 mo/M	Left	VSD	DR, VSD repair	Survived	Alive at 2 yr
2004	Razavi	40 yr/M	--	PAVSD, double aortic arch	Conservatively managed	N/A	Assessed for transplant
2005	Vida	13 Dy/--	Right	TOF, 22-q11 microdeletion	DR, VSD Repair, RVOT Repair	Survived	LPA stenosis at 48 mo
	Vida	9 wk/--	Right	TOF, 22-q11 microdeletion	DR	Survived	Alive at 104 mo
2005	Carretero	23 Dy/M	Right	TOF, 22 q11 microdeletion	DR, VSD repair	Survived	Alive
2006	Nathan	5 mo/--	--	VSD	DR, VSD Repair	Survived	Alive
	Nathan	25 day/--	--	PFO, VSD	DR, VSD Repair	Survived	Alive
2007	Zhang	--	--	--	--	--	--
	Zhang	--	--	--	--	--	--
	Zhang	--	--	--	--	--	--
2008	Bockeria	2 yr/M	--	DORV, AORSA,	DR, DORV repair	Survived	Alive at 4 yr
2008	Cheng	10 yr/M	--	TOF	DR, TR	Survived	Alive at 3 mo
2008	Li	33 m/F	--	VSD	DR, VSD repair	Survived	Alive at20 mo
2010	Amir	11 days	Right	PFO, R-PDA	DR	Survived	Alive
	Amir	6 mo	Right	R-PDA	DR	Survived	Alive
2010	Khositseth	10 mo/M	Right	PAVSD, MAPCA-RL	Staged Repair shunt LPA-Ao	--	--
2010	Diab	2 mo/--	Right	TOF	DR, TR	Survived	Alive at 1 yr
2010	Erdem	--	--	--	DR	Survived	--
2010	Goldstein	--	--	--	--	--	--
	Goldstein	--	--	--	--	--	--
2011	Pepeta	10 mo/M	Left	PAVSD, ARSA, MAPCA-RL	Conservatively managed	N/A	--
	Pepeta	6 yr/F	Left	PAVSD, MAPCA-RL	Conservatively managed	N/A	--
2011	Sun	2 yr/M	--	TOF	DR, TR	Survived	Alive at 1 yr
2011	Dwivedi	12 yr/M	Right	TOF	DR, TR	Survived	Alive at 6 mo
2012	Aly	13 day/F	Right	TOF, APV	DR, TR	Survived	Alive at10 mo
2012	Garg	1 yr/M	Left	VSD, ASD	DR, VSD, ASD closure	Survived	Alive at 2 mo
	Garg	7 mo/M	Left	TOF	DR, RPA shunt—without CPB	Died	Died
	Garg	6 yr/F	Left	TOF	DR, TR	Survived	Alive at 2 mo
	Garg	13 yr/F	Right	TOF	DR, TR	Survived	Alive at 2 mo
2013	Haddadin	2 mo/M	Right	None-Isolated	DR	Survived	Alive at 4 wk
2013	Tantiwongkorsi	9 yr/M	Right	TOF	DR, VSD closure	Survived	--
2013	Tsukimori	36 day/F	Left	TOF, APV	TR, RPA plication LPA reconstruction	Survived	Alive
2013	Sanz	6 mo/F	Right	VSD	DR	Survived	Alive at 6 mo
2014	Nigam	19 yr/M	Right	None-Isolated	--	--	--
2014	Talwar	7 mo/M	Left	TOF	DR, TR	Died	Severe Low CO Postop
	Talwar	72 mo/F	Left	TOF	DR, TR	Survived	Alive at18 mo
	Talwar	156 mo/F	Right	TOF	DR, TR	Survived	Alive at56 mo
	Talwar	42 mo/M	Right	TOF	DR, TR	Survived	Alive at 3 mo
	Talwar	33 mo/F	Left	TOF	DR, TR	Survived	Alive at 6 mo
	Talwar	7 mo/M	Left	TOF	DR without CPB	Died	Post op cardiac arrest
2014	Mathur	16 yrF	Left	TOF	TR, DR	Survived	Alive
2015	Liu	--	Right	TOF	--	--	--
	Liu	--	Right	VSD	--	--	--
2015	Paredes	6 wk/M	Right	TOF, 22 q11 microdeletion	DR	Survived	TR at 6 mo
2015	Akyuz	21 Dy/f	Left	None-Isolated	DR, without CPB	Survived	Alive, Post op pneumonia
2015	Cho	2 yo	--	TOF, APV, PFO	DR, VSD repair monocusp implantation	Survived	PVR, LPA Angioplasty-14 yr postop
	Cho	3 mo	--	PDA, PFO	PFO closure, PDA Ligation, DR	Survived	Alive
	Cho	10 mo	--	VSD, PDA, PFO	PFO/VSD closure PDA Ligation, DR	Survived	Alive
	Cho	28 Dy	--	TOF, APV, ASD	DR, TR, ASD rep	Survived	Alive
2015	Nicholson	34 wk gestation	Left	DORV, VSD	DR	--	--
2015	Vasquez	1 yr/M	--	None-Isolated	No CPB, DR	Survived	Alive at 6 mo
	Vasquez	7 yr/F	--	None-Isolated	No CPB, DR	Survived	Alive at 2 mo
2015	Selcuk	13 yr/F	Right	Right pulmonary artery atresia	Conservatively managed	N/A	Treat pulmonary infections
2017	Hussain	1 mo/F	Right	PFO	DR	Survived	Alive at publication
2017	Loomba (Current Study)	34 wk Gestation	Right	ALSA	None	Died	*E. Coli* Sepsis with necrotizing enterocolitis, support withdrawn

**Table 2:** Associated anomalies in addition to anomalous origin of left pulmonary artery from ascending aorta. AR(L)SA = Aberrant Right (Left) Subclavian Artery, DORV = Double Outlet Right Ventricle, MAPCA-RL= Major Aortopulmonary Collateral Vessels Supplying the Right Lung, TOF = Tetralogy of Fallot, VSD = Ventricular Septal Defect.

	Associated Anomalies
Total Reported	113
TOF (All Instances)	52.2% (59)
TOF, APV	7.9% (9)
Isolated	13.3% (15)
R-PDA	14.6% (16)
22q11.1	7.1% (8)
VSD (non-TOF)	17.7% (20)
ALSA	4.5% (5)
ARSA	2.7% (3)
MAPCA-RL	4.4% (5)
DORV	2.7% (3)
Adult	4.4% (5)
Arch Reported	74
Right	68.9% (51)
Left	31.1% (23)

**Table 3:** Causes of mortality and age of death by case. AR(L)SA = Aberrant Right (Left) Subclavian Artery, CPB = Cardiopulmonary Bypass, DR = Direct Reimplantation, MOF = Multi-organ Failure, PAVSD = Pulmonary Atresia Ventricular Septal Defect, PDA = Patent Ductus Arteriosus, PVOD = Pulmonary Vascular Obstructive Disease, RL = Right Lung, TOF = Tetralogy of Fallot, TR = Tetralogy of Fallot repair, VSD = Ventricular Septal Defect. Dy = days, mo = months, yr = years, wk = weeks.

Year	Author	Age at Death	Defect	Surgical Procedure	Notes
1941	Thomas	3 mo	TOF	None	Bronchopneumonia
1952	Sikl	Stillborn	TOF	None	None
1964	Czarneck	4 yr	TOF	VSD repair, TR	Unable to wean from CPB
1975	Calazel	14 mo	TOF	TR	None
1978	Brill	3 mo	PDA	None	Died 30 min after admission, upper respiratory tract infection
1974	Keane	4 mo	TOF, bi-lobed RL	None	Postmortem Finding
1975	Robin	5 mo	TOF	Conservatively Managed	Died 1 h after admission, fever and cyanosis,
1978	Sotomora	Newborn	TOF, R-PDA, ALSA	None	Postmortem finding
1980	Calder	1 mo	TOF/APV	None	Died 4 h after admission, inhalation pneumonia
	Calder	10.5 mo	TOF/APV	Conservatively Managed	Discharged, Died 8.5 mo later from “a chest cold”
1985	Robida	4 yr	TOF	DR, TR	Died immediate postop, advanced pulmonary vascular disease
1989	Fong	26 mo	TOF	DR, TR	High RV pressure, poor CO, severe vascular changes on left lung
1990	Cherian	5 yr	TOF	TR	None
1992	Endo	13 yr	TOF	DR, TR	Died 38 th day post op, Low cardiac output syndrome, PVOD
1996	Tagliente	56 Dy	None	DR	Died post op day 32, MOF
1999	Matsubayashi	3 mo	VSD, RPA-dAO	VSD repair, Reconstruction	Died post op Day 4
2012	Garg	7 mo	TOF	DR without CPB	Died post op
2014	Talwar	7 mo	TOF	DR without CPB	Died 1 day post op, Severe low cardiac output syndrome
2014	Talwar	7 mo	TOF	DR without CPB	Died 6 h post op, cardiac arrest
2017	Loomba	34 wk Gest.	ALSA	Support Withdrawn	E. Coli Sepsis with necrotizing enterocolitis

## Data Availability

The raw data is available upon request.
